# Testing the consequences of alcohol, cannabis, and nicotine use on hippocampal volume: a quasi-experimental cotwin control analysis of young adult twins

**DOI:** 10.1017/S0033291721004682

**Published:** 2021-11-17

**Authors:** Jeremy Harper, Sylia Wilson, Jessica L. Bair, Ruskin H. Hunt, Kathleen M. Thomas, Stephen M. Malone, William G. Iacono

**Affiliations:** 1 Department of Psychiatry & Behavioral Sciences, University of Minnesota, Twin Cities, USA; 2 Institute of Child Development, University of Minnesota, Twin Cities, USA; 3 Veterans Affairs Ann Arbor Healthcare System, Ann Arbor, USA; 4 Department of Psychology, University of Minnesota, Twin Cities, USA

**Keywords:** Alcohol, cannabis, cotwin control analysis, hippocampal volume, nicotine, sex differences, substance use

## Abstract

**Background.:**

Alcohol, cannabis, and nicotine use are highly comorbid and alarmingly prevalent in young adults. The hippocampus may be particularly sensitive to substance exposure. This remains largely untested in humans and familial risk may confound exposure effects. We extend prior work on alcohol and hippocampal volume in women by testing common and unique substance use effects and the potential moderating role of sex on hippocampal volume during emerging adulthood. A quasi-experimental cotwin control (CTC) design was used to separate familial risk from exposure consequences.

**Methods.:**

In a population-based sample of 435 24-year-old same-sex twins (58% women), dimensional measures (e.g. frequency, amount) of alcohol, cannabis, and nicotine use across emerging adulthood were assessed. Hippocampal volume was assessed using MRI.

**Results.:**

Greater substance use was significantly associated with lower hippocampal volume for women but not men. The same pattern was observed for alcohol, cannabis, and nicotine. CTC analyses provided evidence that hippocampal effects likely reflected familial risk and the consequence of substance use in general and alcohol and nicotine in particular; cannabis effects were in the expected direction but not significant. Within-pair mediation analyses suggested that the effect of alcohol use on the hippocampus may reflect, in part, comorbid nicotine use.

**Conclusions.:**

The observed hippocampal volume deviations in women likely reflected substance-related premorbid familial risk and the consequences of smoking and, to a lesser degree, drinking. Findings contribute to a growing body of work suggesting heightened risk among women toward experiencing deleterious effects of substance exposure on the still-developing young adult hippocampus.

## Introduction

Alcohol, nicotine, and other substance use is a leading public health concern among young adults aged 18–25 (i.e. the developmental period referred to as emerging adulthood). Rates of substance use are alarmingly high among young adults; recent estimates from a 2019 United States national survey (Substance Abuse and Mental Health Services Administration, 2020) indicate that 34% engaged in past-month binge drinking, 24% had used tobacco products in the past month (one-third of which were daily cigarette smokers), and nearly 35% used cannabis in the past year. While substance use rates among young adults were on the decline in the early twenty-first century, recent estimates suggest that this is no longer the case (Substance Abuse and Mental Health Services Administration, 2020). High levels of substance exposure during emerging adulthood occur alongside continued cortical and subcortical structural development (Shaw et al., 2008; Sowell et al., 2003; Wierenga, Langen, Oranje, & Durston, 2014), including the hippocampus (Wierenga et al., 2014), which may create a developmental period of vulnerability where substance exposure may impact the still-developing young adult brain.

Evidence strongly indicates that the hippocampus is involved in learning and memory and the hippocampus has been implicated in neural circuitry models of substance use/addiction (Koob & Volkow, 2010, 2016) playing a role in drug-related contextual processing and anticipation/craving. In human neuroimaging studies, lower hippocampal volume is associated with greater use of alcohol (Fein & Fein, 2013; see Wilson, Bair, Thomas, & Iacono, 2017 for a meta-analysis), cannabis (Filbey, McQueeny, Kadamangudi, Bice, & Ketcherside, 2015; Yücel et al., 2008; see Rocchetti et al., 2013 for a meta-analysis), nicotine (Janowitz et al., 2014) and other illicit substances (Thompson et al., 2004). While most studies offer evidence in support of lower hippocampal volume in relation to substance use (Mackey et al., 2018), some recent studies report null associations (Filbey et al., 2015; Gillespie et al., 2018; Mashhoon, Sava, Sneider, Nickerson, & Silveri, 2015). These discrepancies may be attributed to various potential limitations, including small sample sizes (*Ns*~90; as noted by Gillespie et al., 2018), use of coarse substance use measures (e.g. users *v*. non-users) that limit statistical power, or failing to consider sex-specific effects. There is emerging evidence that women, a traditionally under-represented population in substance use research (Verplaetse, Cosgrove, Tanabe, & McKee, 2021; Zilverstand, Huang, Alia-Klein, & Goldstein, 2018), may be at heightened risk of experiencing substance-related adverse outcomes or exposure-related consequences relative to men (Becker & Koob, 2016; Erol & Karpyak, 2015; Seo et al., 2019; Wilhelm et al., 2016). As many prior reports only evaluated one substance, it also remains unclear if hippocampal deviations reflect substance use in general (that is, observed across many substances), comorbid substance use, or only certain forms of substance use.

The hippocampus is a cannabinoid and nicotinic acetylcholine receptor (nAChR) dense region and is one of the only brain regions known to exhibit adult neurogenesis (Vadodaria & Jessberger, 2014). Preclinical rodent work suggests that hippocampal structure and adult neurogenesis may be particularly susceptible to substance exposure effects, including alcohol (Nixon & Crews, 2002), cannabis (Prenderville, Kelly, & Downer, 2015; Rusznák et al., 2018), and nicotine (Abrous et al., 2002; Csabai et al., 2016; as reviewed in Canales, 2007), which in turn may affect continued substance use (Mandyam & Koob, 2012). Translating animal models of substance use to humans is a difficult task and determining whether substance-related hippocampal volume deviations in humans reflect the consequence of substance exposure remains a major challenge because of potential confounding from familial risk (e.g. genetic liability, rearing environment). While longitudinal studies can establish a temporal sequence between an exposure and an outcome, such as greater hippocampal decline between assessments in heavy drinkers (Meda et al., 2018) or smokers (Duriez, Crivello, & Mazoyer, 2014), or lower hippocampal volume predicting binge drinking (Whelan et al., 2014), longitudinal studies of genetically unrelated individuals are not immune from familial confounding.

The cotwin control (CTC) design (Carlin, Gurrin, Sterne, Morley, & Dwyer, 2005; McGue, Osler, & Christensen, 2010) is a ‘natural’ quasi-experiment that approximates true experiments (Rubin, 2007; Thapar & Rutter, 2019) by relating naturally occurring variations in exposure within twin pairs to differences in an outcome. Twin differences in exposure control for familial risk confounding to more appropriately and stringently evaluate potential causal substance exposure effects (independent of familial risk) than is possible with cross-sectional or longitudinal studies of genetically unrelated individuals. In this design, the hippocampal volume of a lesser-using twin provides an estimate of the expected volume for their heavier-using cotwin had s/he used less; if smaller volume reflects an exposure effect, the heavier-using twin is expected to have lower volume relative to their lesser-using cotwin after adjusting for familial risk confounding. This approach was used in a recent paper from our group which provided evidence in support of alcohol use negatively impacting hippocampal-mediated learning performance (Malone, Wilson, Bair, McGue, & Iacono, 2021).

The current study was designed to address the aforementioned gaps in the literature and expand on our prior work using the CTC to study the association between alcohol use, familial risk, and hippocampal volume in women that was examined by Wilson, Malone, Hunt, Thomas, and Iacono (2018) in a study of 100 24-year-old female twins. Wilson et al. (2018) found that drinking was associated with reduced hippocampal volume, and CTC analyses were consistent with reduced volume reflecting the consequence of alcohol use.

Here we extend our previous pilot study to test the causal relationship between alcohol, cannabis, and nicotine use during emerging adulthood and hippocampal volume in a much larger (*N* = 435) population-based, etiologically informative twin sample that includes women and men. Dimensional quantitative measures of substance use were assessed to capture variation in normative patterns (i.e. none to heavy) of use/exposure across emerging adulthood. First, we hypothesized a negative phenotypic association between (poly)substance use (a composite measure of alcohol, cannabis, and nicotine use) and hippocampal volume and tested whether sex moderated this effect to test whether substance-related effects were stronger in women than men. Significant phenotypic effects for substance use, in general, were followed up to explore potential differential associations between alcohol, cannabis, or nicotine use and hippocampal volume deviations. For all significant phenotypic associations, the CTC design was used to disentangle exposure-related effects from familial risk on hippocampal volume. Because substance use is often comorbid and the CTC cannot account for unshared confounders, follow-up mediation analyses in a within-pair multilevel framework (Zhang, Zyphur, & Preacher, 2008) were conducted to test whether within-pair effects for a particular substance were independent or due to confounding from co-occurring twin differences in the use of other substances.

## Methods

### Sample

Participants were same-sex twins assessed at the target age of 24 years from the population-based Minnesota Twin Family Study Enrichment Sample (Keyes et al., 2009). By design (e.g. participants met standard MRI safety criteria and could complete in-person MRI assessment, etc.), 441 individuals completed structural MRI scans. Four individuals with clinically significant brain anomalies (determined by a clinical radiologist) and one with scanner coil failure were excluded from the analysis. Diagnostic examination of the statistical models using the outlierTest function in the *car* R package (Fox & Weisberg, 2019) identified one individual whose data was an outlier in all linear mixed models (∣Studentized residuals∣ ⩾4.02, Bonferroni-adjusted *p* values ⩽0.0186;); data for this individual was excluded from all analyses. The final sample included 435 individuals [age: mean (s.d.) = 24.3 (0.8) years; 253 women; racial composition: 92.2% White/Caucasian, 2.8% Black/African American; 2.5% Hispanic; 1.4% mixed/other; 0.7% Native American; 0.5% Asian/Pacific Islander], with 120 complete MZ pairs (i.e. 240 MZ twins), 30 unpaired MZ twins, 65 complete DZ pairs (130 DZ twins), and 35 unpaired DZ twins. The zygosity by sex breakdown was as follows: men: 51 complete MZ pairs, 15 unpaired MZ twins, 22 complete DZ pairs, and 21 unpaired DZ twins; women: 69 complete MZ pairs, 15 unpaired MZ twins, 43 complete DZ pairs, and 14 unpaired DZ twins. 100 women in this sample were included in our previous report (Wilson et al., 2018).

### Substance use assessment

Substance use was assessed with an expanded version of the Substance Abuse Module of the Composite International Diagnostic Interview (Robins, Babor, & Cottler, 1987) administered by trained interviewers.

A drink index (possible range: 0.00–5.75) was constructed by averaging four alcohol use items: frequency of drinking (last 7 years); a typical number of drinks per occasion (amount; last 7 years); the maximum number of drinks drank in 24 h (last 7 years); and a number of intoxications (lifetime) [Cronbach’s *α* = 0.78; average pairwise *r* = 0.47 (range: 0.22–0.61)]. A cannabis index (possible range: 0.00–5.00) was calculated by averaging two cannabis use items (last 7 years): frequency; a number of uses (pairwise *r* = 0.94). Cigarettes per day (during a period of heaviest use in last 7 years), adjusted for nondaily use and, when applicable, equivalent use of other tobacco products (e.g. chew, cigars), was calculated according to our previous report (Elkins et al., 2018); possible scores on this measure ranged from 0 (none) to 4 (⩾20 cigarettes, equivalent to a pack or more per day). See online Supplementary Table S1 for further details.

To obtain a measure of composite substance use, the drink index, cannabis index, and cigarettes per day scores (pairwise *r* range = 0.47–0.57; Cronbach’s *α* = 0.77) were standardized and then averaged.

### Neuroimaging acquisition and processing

Structural MRI data were collected on 3 T Siemens Trio (*n* = 100) and Prisma (*n* = 336) MRI scanners (32-channel array head coil) at the Center for Magnetic Resonance Research, University of Minnesota. A scanner software upgrade occurred mid-study (*n*: pre-upgrade = 306, post-upgrade = 130). Three-dimensional T1-weighted sagittal plane anatomical images were acquired using the following magnetization prepared rapid gradient echo sequence: TR = 2530 ms; TE = 3.65 ms; flip angle = 7°; matrix size = 256 × 256; FOV = 256 mm; GRAPPA = 2; 240 coronal slices with 1-mm isotropic voxels; single shot; interleaved acquisition. Images were normalized and manually reviewed for artifacts/structural anomalies and processed using the Freesurfer pipeline (version 5.3.0; Fischl et al., 2002, 2004). Volumetric data were obtained for the left and right hippocampus and an estimate of total brain volume (‘BrainSegVolNotVent’) was calculated.

### Statistical analysis

All statistical analyses were done in R (R Core Team, 2019) using linear mixed models (LMMs; *lme4* package; Bates, Machler, Bolker, & Walker, 2015) with random intercepts at the family level to adjust for within-pair correlations in dependent variables. LMMs included sex, age, zygosity, scanner, acquisition software, and total brain volume as covariates. To provide an estimate of uncertainty around effect sizes and determine significance, the *bootmlm* package (Lai, 2020) was used to conduct nonparametric residual bootstrapping of the LMMs (5000 random draws; clustered by family) to calculate bootstrap estimates of standard errors, and the *boot* package (Canty & Ripley, 2021) was used to compute bias-corrected and accelerated 95% confidence intervals (CIs) around the fixed effect unstandardized beta estimates (for a technical discussion, see Carpenter, Goldstein, & Rasbash, 2003; Leeden, Meijer, & Busing, 2007). A parameter estimate was considered significant if the bootstrapped CI did not contain zero. As left and right hippocampal volumes were highly correlated (*r* = 0.81) and we had no a priori hypothesis regarding laterality effects, to reduce Type I error rate total hippocampal volume scores (summed across left/right hemispheres) were used in all primary analyses. Supplementary analyses were conducted to test whether effects differed by hemisphere.

First, an LMM was fit to test the individual-level phenotypic association between hippocampal volume (dependent measure) and the substance use composite (independent measure); sex (coded for women) by substance use interaction term tested whether the substance use composite effect was moderated by sex and greater in women than men. Significant hippocampus-substance use composite effects were followed up with separate models testing the association between hippocampal volume and drink index, cannabis index, and cigarettes per day scores to evaluate whether observed effects were driven by general substance use (i.e. common to all three substances) or a particular substance.

For significant individual-level associations, follow-up CTC analyses (McGue et al., 2010) tested causal exposure and familial risk effects by treating twins as genetic and shared environmental controls to adjust for all sources of familial influence confounded with the exposure (e.g. alcohol use). In this design, hippocampal volume was compared between cotwins; if a twin had a higher level of substance use relative to her/his cotwin, the outcome (i.e. hippocampal volume) of the lesser-using twin provided a close approximation of the expected outcome for the heavier-using twin had she/he used less (the unobserved counterfactual case; Rutter, 2007). In the CTC analysis, the substance use score was separated into two orthogonal components: (1) the between-pair effect, expressed as the twin-pair mean score, indexing all genetic and shared environmental familial risk influences, whether measured or unmeasured; and (2) the within-pair effect, expressed as an individual twin’s difference from their twin-pair mean score, reflecting twin differences in substance use and the nonshared environmental influence of substance exposure on an outcome (Begg & Parides, 2003). Distributions of the scores used in the CTC models are shown in online Supplementary Fig. S1. LMMs were fit with hippocampal volume as the dependent variable and the between-pair and within-pair terms as independent fixed effects using complete twin pairs (unpaired twins were excluded from the CTC). A significant between-pair effect would be consistent with familial risk influencing both alcohol use and hippocampal volume. In contrast, a significant within-pair effect would be consistent with the interpretation that substance exposure (unconfounded by all familial factors influencing use) confers a potential effect on hippocampal volume (e.g. heavier-using twins exhibiting decreased volume compared to lesser-using cotwins). For significant within-pair effects, we compared the strength of the within-pair effect between MZ (100% genetic control) and DZ (50% genetic control) twin pairs using zygosity by within-pair interaction; statistically comparable MZ/DZ within-pair effects are considered strong evidence consistent with an exposure effect (McGue et al., 2010).

Because the CTC cannot control for unshared confounders, follow-up mediation analyses in a within-pair multilevel framework (Zhang et al., 2008) were conducted to evaluate whether any observed within-pair effects were due to (partial) confounding from twin differences in co-occurring substance use. This approach is particularly useful to test alternative causal models in the CTC framework when more than one within-pair effect is significant to determine if an observed within-pair effect for one substance is because of twin differences in another substance (Malone et al., 2021).

## Results

### Descriptive statistics

Descriptive statistics are presented in Table 1. Drink index, cannabis index, and cigarettes per day scores were moderately correlated (pairwise correlations: 0.47–0.57); all were highly correlated with the substance use composite (pairwise correlations: 0.82–0.86). The use of all substances was greater among men relative to women. While raw hippocampal volume (unadjusted for total brain volume) was smaller in women than men [Beta (95% CI) = −661.05 (−860.84 to −472.27), s.e. = 98.79], this difference was diminished after adjusting the hippocampal volume for total brain volume [Beta (95% CI) = −97.48 (−280.92 to 89.13), s.e. = 94.42; least-squares adjusted means: women: 8219 mm^3^, men: 8317mm^3^].

### Individual-level phenotypic associations

**Fig. 1.**
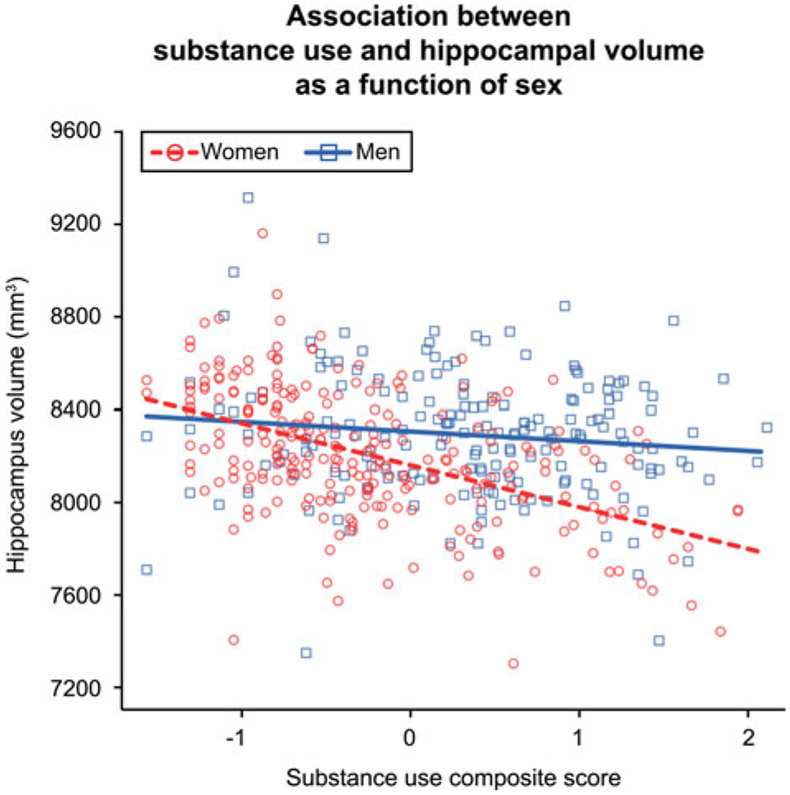
Plot depicts the individual-level phenotypic interaction between substance use composite scores and sex. Lines represent the linear mixed model fit lines for the interaction term. Greater substance use was associated with significantly lower hippocampal volume in women but not men. The *visreg* R package (Breheny & Burchett, 2017) was used to create the partial residual plot.

Hippocampal volume had a significant negative association with substance use composite scores [Beta (95% CI) = −122.80 (−193.88 to −53.51), s.e. = 35.75]. This effect was qualified by an interaction between substance use composite scores and sex [Beta (95% CI) = −58.21 (−116.53 to −2.77), s.e. = 29.38], as illustrated in Fig. 1. The interaction reflected the significant negative relationship between hippocampal volume and substance use observed in women [Beta (95% CI) = −184.99 (−270.10 to −95.90), s.e. = 44.77] and the negligible/non-significant effect for men [Beta (95% CI) =−33.30 (−151.34 to 75.66), s.e. = 57.05]. This suggested that the substance use – hippocampus effect was moderated by sex and driven by the negative association observed in women; further analyses were conducted separately by sex to investigate the associations between hippocampal volume and alcohol, cannabis, and cigarettes per day.

As reported in Table 2, for women, the same pattern of effects was observed across all substances. Mirroring the substance use composite effect, greater alcohol use, cannabis use, and cigarettes per day were significantly associated with lower hippocampal volume in women. In contrast, effects for men were again small and non-significant.

Finally, to determine whether specific forms of substance use accounted for unique phenotypic variance in hippocampal volume above and beyond that shared across substances, a single model including the three separate phenotypes (drink index, cannabis index, cigarettes per day) as independent variables was constructed for women. When tested together, the drink index [Beta (95% CI) = −100.72 (−180.88 to −25.37), s.e. = 39.95] and cigarettes per day [Beta (95% CI) = −97.40 (−168.63 to −24.75), s.e. = 36.72] terms were significant, whereas the cannabis index term was not [Beta (95% CI) = 15.78 (−32.76 to 72.75), s.e. = 26.73], suggesting that alcohol/nicotine explain unique variance in hippocampal volume above and beyond that shared among all three substances.

### Cotwin control analysis (CTC)

The CTC analysis was used to separate familial risk influences from deleterious environmental consequence effects for the significant negative associations between substance use composite, drink index, and cigarettes per day scores on total hippocampal volume in women (112 complete twin pairs).

**Fig. 2.**
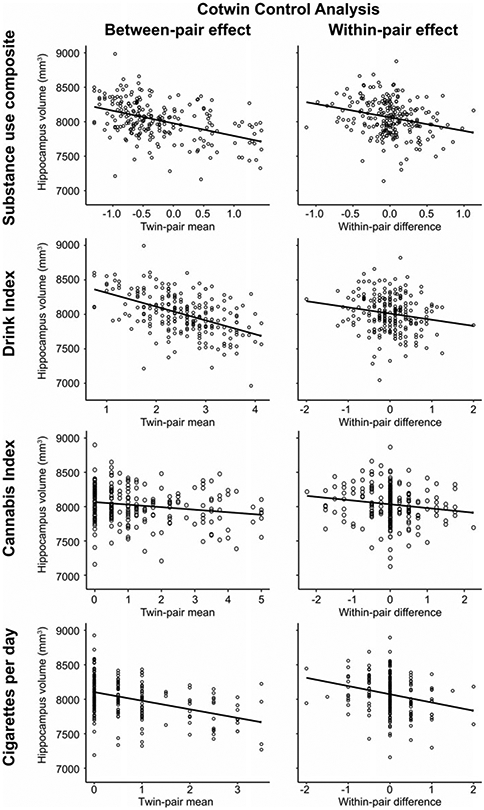
Plots depict the cotwin control analysis models of substance use on total hippocampal volume in women. Between-pair and within-pair effects are depicted with model fit lines from the linear mixed models reported in Table 3. The between-pair effect plots illustrate the significant association between lower hippocampal volume and the mean level of substance use composite, drink index, or cigarettes per day scores within a twin pair, consistent with a premorbid familial risk association. The within-pair effect plots illustrate that heavier-using twins (positive within-pair difference scores), and in particular, the heavier-drinking and heavier-smoking twins, exhibited significantly lower hippocampal volume relative to their lesser-using cotwins (negative within-pair difference scores), consistent with an exposure effect. The cannabis between-pair and within-pair effects had the expected negative association with hippocampal volume but neither effect was significant. The *visreg* R package (Breheny & Burchett, 2017) was used to create the partial residual plots.

Results of the CTC analysis are reported in Table 3 and depicted in Fig. 2. For the substance use composite, the between-pair effect, representing familial influences shared by twins, and the within-pair effect, reflecting nonshared environmental influence unconfounded by all shared familial influences, had significant negative associations with hippocampal volume. Turning to the individual substance use phenotypes, the same pattern of significant between-pair and within-pair effects were observed for the drink index and cigarettes per day. That is to say that, after accounting for all sources of familial risk, twins with greater substance use, and in particular, those who used more alcohol or nicotine, had lower hippocampal volume relative to their lesser-using cotwins, consistent with an exposure-related consequence. Consistent with this interpretation, the within-pair effects between MZ and DZ twin pairs were statistically equivalent [in all cases, including zygosity by within-pair interaction term did not significantly improve model fits, ($\Delta \chi_{\left(1\right)}^{2}$ ⩽1.16, *p*s ⩾ 0.281) and the interaction terms were non-significant (all 95% CIs contained 0)]. While in the expected negative direction, neither between- nor within-pair effects were significant for the cannabis index.

The CTC design accounts for all shared confounders but cannot control for individual-specific factors that differ between cotwins and may confound a within-pair effect. For example, the effect of drinking on hippocampal volume could be due to confounding from twin differences in smoking if heavier-drinking twins are more likely to smoke than their lesser-drinking cotwins. The drink index and cigarettes per day within-pair difference scores used in the CTC reported above were moderately correlated (*r* = 0.23), raising this possibility. To address the potential for unshared confounding, we assessed within-pair mediation (Zhang et al., 2008) by including the drink index and cigarettes per day within-pair difference scores as joint predictors of hippocampal volume in the CTC to evaluate an alternative causal model – i.e. that the observed drink index within-pair effect on hippocampal volume was actually due to the effect of co-occurring twin differences in cigarette smoking (or vice versa).

The within-pair mediation analysis indicated that the cigarettes per day within-pair effect on hippocampal volume was slightly reduced yet remained significant [Beta (95% CI) = −103.95 (−184.39 to −13.13), s.e. = 44.02], whereas the drink index within-pair effect did not [Beta (95% CI) = −65.83 (−152.27 to 16.27), s.e. = 42.84]. This suggests that the drink index within-pair effect is in part attributable to co-occurring nicotine use, while the cigarettes per day within-pair effect was independent and robust to comorbid alcohol use.

## Discussion

The present study significantly expanded on our prior work examining the causal association between alcohol and hippocampal volume in women (Wilson et al., 2018) by evaluating possible casual associations between hippocampal volume and alcohol, cannabis, and nicotine use during emerging adulthood in a large population-based sample of 24-year-old twins that included men and women. Within neural circuitry models of substance use/addiction (Koob & Volkow, 2010, 2016), the hippocampus is thought to play a crucial role in drug-related contextual processing and anticipation/craving. Lower hippocampal volume has been associated with greater substance use in humans (Mackey et al., 2018; Wilson et al., 2017). While animal work offers evidence that the hippocampus may be particularly susceptible to the effects of alcohol, cannabis, and nicotine exposure (Canales, 2007; Csabai et al., 2016; Nixon & Crews, 2002; Rusznák et al., 2018), the nature of the substance use-hippocampal volume association in humans has been largely unclear given difficulties disentangling familial risk from environmental effects in observational research. Using a ‘natural’ quasi-experimental CTC design to strengthen causal inferences, this study provides new evidence that lower hippocampal volume in women likely reflects both the brain-based expression of substance use familial risk and the potential deleterious environmental consequence of nicotine, and to a lesser extent, alcohol use, on the young adult hippocampus.

Using dimensional measures to index salient characteristics of substance use/exposure across the emerging adulthood period, we observed sex-specific associations at the phenotypic level where greater composite substance use, a measure collapsing across alcohol, cannabis and nicotine use, was associated with lower hippocampal volume in women but not men. Lower hippocampal volume in women was also observed for greater use of alcohol, cannabis, and nicotine individually. This is consistent with prior work demonstrating negative associations with hippocampal volume across many forms of substance use (Fein & Fein, 2013; Mackey et al., 2018; Wilson et al., 2018; Yücel et al., 2008) and substance use comorbidity (e.g. nicotine and marijuana; Filbey et al., 2015), and suggests that substance-related individual differences in hippocampal volume are sensitive to the propensity toward substance use in general rather than one substance exclusively. While all three substance use measures showed similar associations, only alcohol and nicotine use explained independent variance in hippocampal volume. Co-occurring alcohol and nicotine use reflects the most common form of polysubstance use, and the present findings may help contribute to our understanding of the neurobiology of comorbid alcohol and nicotine use (Van Skike et al., 2016).

The observed associations between substance use measures and hippocampal volume were moderated by sex, such that significant negative associations were observed for women but not men. Many prior substance use studies vastly under-sampled or excluded women from the sample (Verplaetse et al., 2021; Zilverstand et al., 2018), and the current study sought to address the important gap in the field regarding potential sex-specific effects for brain outcomes and substance use. The present results add to a growing body of literature suggesting that women may be at heightened risk of exhibiting or developing deleterious substance-related outcomes relative to men (Becker & Koob, 2016; Wilhelm et al., 2016) including physical health problems (Erol & Karpyak, 2015), worse neurocognitive performance (Nolen-Hoeksema, 2004), prefrontal brain electrophysiology deviations (Harper, Malone, & Iacono, 2018), and neuroanatomical variations (Seo et al., 2019; Welch, Carson, & Lawrie, 2013). While specific biological mechanisms of this suspected heightened vulnerability are still unclear, as discussed by Erol and Karpyak (2015), this may be due to sex differences in pharmacokinetics (e.g. metabolism) or interactions between sex hormones and substance use. Recent experimental work suggests that accelerated changes in hippocampal plasticity have been observed in female but not male rodents at similar doses of ethanol, an effect driven by the presence of high estrogen levels in female rats (Rabiant, Antol, Naassila, & Pierrefiche, 2021). Evidence also suggests that some nicotine-related sex differences may be mediated by the influence of sex hormones on nicotine metabolism or craving and contextual processing/reinforcement (Cross, Linker, & Leslie, 2017), themselves processes closely related to the hypothesized involvement of the hippocampus in neural circuitry models of substance use/addiction (Koob & Volkow, 2010, 2016). Further research should consider the possible importance of substance-related sex differences.

Evidence from the CTC analysis, utilizing twins as ideal genetic and shared environmental controls, suggests that lower hippocampal volume partially reflects the familial vulnerability toward greater substance use. Significant between-pair effects were observed for the substance use composite, drink index, and cigarettes per day. This pattern is consistent with the phenotypic-level analyses discussed above and offers evidence that individual differences in hippocampal volume are premorbid characteristics that may confer risk for polysubstance use, and specifically, alcohol and nicotine use. In this sample, the cannabis index between-pair effect was in the expected direction but not significant whereas the risk effect was stronger for alcohol and nicotine. This may be due to differences in the polygenic underpinnings of alcohol, nicotine, and cannabis use, which have moderate but not complete genetic overlap (Jang et al., 2020; Liu et al., 2019). Substance use is comorbid with other externalizing behaviors (Iacono, Malone, & McGue, 2008; Krueger & Markon, 2006), and individuals with this lower hippocampal predisposition may be at higher vulnerability for other related negative outcomes including addiction, conduct disorder/antisocial behavior, etc. The present study may help to clarify the relationship between familial risk and hippocampal deviations. Prior work in adolescents using the high-risk offspring design has been mixed on whether a family history of substance use disorder (primarily alcohol use disorder) is associated with structural hippocampal deviations (for narrative reviews, see Comstock, Vaidya, & Niciu, 2019; McPhee et al., 2018). The CTC design used in this report is an alternate, more stringent, approach for separating risk from exposure (McGue et al., 2010; Rutter, 2007; Thapar & Rutter, 2019), and future work using this design may help shed further light on associations between substance use familial risk and hippocampal volume deviations.

In addition, CTC results suggested a deleterious potential exposure-related consequence (within-pair effect) of composite substance use, drink index, and cigarettes per day exposure on lower hippocampal volume in women. Comparing members of twin pairs discordant in their levels of substance use, and particularly alcohol and nicotine, which controls for all sources of shared familial confounding shared by members of a twin pair, lower hippocampal volume was observed in women who used more heavily relative to their lesser-using cotwins. Again, while in this sample the cannabis within-pair effect was in the expected negative direction, its CI spanned zero. Despite the relatively high density of cannabinoid receptors in the hippocampus, the potential exposure effects of cannabis use on hippocampal volume in this sample are weaker than the exposure effects observed for nicotine and alcohol. Because the CTC cannot control for unshared confounding, and twin differences in drinking were positively correlated with twin differences in cigarette smoking, a within-pair mediation approach (Zhang et al., 2008) was utilized to test if the observed within-pair effects for alcohol and nicotine were independent or confounded by co-occurring twin differences in the use of the other substance. Adjusting the drink index and cigarettes per day within-pair effects for each other, only the cigarettes per day effect remained significant, suggesting that a portion of the alcohol exposure effect is attributable to the exposure effect of co-occurring nicotine use.

The CTC can offer evidence that greater substance use, in particular, smoking and to a lesser extent alcohol use, may lead to lower hippocampal volume in women, but we caution that it does not necessarily imply a direct causal mechanism. One potential mechanistic explanation for the observed cigarettes per day within-pair effect is the deleterious effect of nicotine exposure on the hippocampus. One of the most enriched regions in the human brain for nicotinic receptors is the hippocampus (Dome, Lazary, Kalapos, & Rihmer, 2010; Picard et al., 2013), and it is particularly dense with high-affinity nAChR *α*4*β*2 receptors. If nicotine does confer a neurotoxic effect, it may be more likely to occur in nAChR dense areas like the hippocampus. Experimental work offers evidence that nicotine exposure in adult rodents decreases the dendritic length of the hippocampal CA3 subfield (Holliday et al., 2016) and affects hippocampal neurogenesis/plasticity (Abrous et al., 2002; Csabai et al., 2016). In agreement with our finding of an exposure effect of nicotine in women but not men, experimental animal work reviewed by Cross et al. (2017) indicates support for sex differences in the association between nicotine and various phenotypes in adolescent and young adult rodents, with females generally experiencing worse outcomes. These include increased subcortical nAChR nicotine binding, higher plasma levels of nicotine with repeated administration, more severe withdrawal symptoms (thought to be associated with ovarian hormones), greater HPA axis activity and corticosterone release, and enhanced stress/anxiety-like behavior in female rodents (Cross et al., 2017; Dome et al., 2010). Similarly, the drink index within-pair effect observed here may reflect the consequences of alcohol exposure, as alcohol is a neurotoxin at high doses, although as suggested by the within-pair mediation analysis, at least a portion of this effect may be secondary to twin differences in smoking. Interestingly, *α*4*β*2-containing nAChRs are implicated in the rewarding properties of both alcohol and nicotine, and more work is needed at different levels of analysis to understand how *α*4*β*2 rich regions like the hippocampus may be differentially affected by alcohol and nicotine (Van Skike et al., 2016). We also acknowledge that rather than direct neurotoxic consequences, nicotine and alcohol use may be correlated with other negative outcomes, such as increased stress/cortisol levels, deleterious physical/emotional psychosocial effects, or risky behaviors that may, in turn, impact hippocampal volume, although such confounders have been shown to have little impact on the hippocampal associations (Wilson et al., 2018). While confirmation in independent samples is needed, given the prevalence of substance use during emerging adulthood, these preliminary findings that normative levels of substance use, primarily nicotine and alcohol exposure, during emerging adulthood may confer deleterious environmental effects on hippocampal volume in women as early as age 24 have potentially significant public health implications.

Major strengths of the present study include our use of a sample of young adults whose degree of substance use is comparable to that seen in the United States population (Substance Abuse and Mental Health Services Administration, 2020), increasing its potential generalizability. The large sample of women relative to many prior related studies (Verplaetse et al., 2021) allowed us to test for sex differences in the association between substance use and hippocampal volume. The use of a genetically informative twin sample and the CTC design strengthens the causal inferences that can be drawn from observational research relative to typical cross-sectional or prospective studies of unrelated individuals (Rutter, 2007). The CTC provides *evidence for* causality but cannot definitively establish causality or rule out reverse causation (McGue et al., 2010). Causal claims of nicotine and alcohol exposure on the hippocampus can be strengthened by testing how substance use affects change in hippocampal volume over time using prospectively assessed genetically informative samples such as the Adolescent Brain Cognitive Development (ABCD) study (Volkow et al., 2018). Prospective studies can also assess whether hippocampal deviations predate substance use involvement, as would be expected given the between-pair familial risk effects reported here. It is unclear whether a hippocampal volume is related to polygenic scores for substance use, which if true, would strengthen the interpretation that individual differences in hippocampal structure index risk. Measurement error can downwardly bias within-pair effects to a greater degree than phenotypic effects (McGue et al., 2010), meaning that within-pair effects may be underestimated. Measurement error may also affect the precision of estimating the between-pair effect for analyses with small cluster sizes (e.g. twins within pairs) relative to analyses with large cluster sizes (e.g. students within classrooms); for a nuanced discussion of between-pair (contextual) effects, see Begg and Parides (2003). The present sample was initially recruited to reflect the demographics of Minnesota in the target birth years (Wilson et al., 2019) and, like Minnesota during the years the twins were born (1988–1994), is predominantly white/Caucasian. This may limit generalizability depending on the degree to which effects of substance exposure on brain structural characteristics might vary by racial and ethnic group. Future work in more diverse samples, such as ABCD, to which our group at the Minnesota Center for Twin and Family Research has contributed a twin sample (Iacono et al., 2018), is needed to address this complex empirical question. The effect sizes observed in men were small to negligible, with the largest corresponding to an *r* of −0.07 between hippocampal volume and cigarettes per day (compare this to corresponding *r* of −0.24 observed in women); however, we caution against interpreting these findings as evidence that there is no possibility for a meaningful effect in men because, as discussed by Funder and Ozer (2019), small effects (i.e. *r* of ∣ 0.05∣) have the potential to cumulate over time and increase in consequence. Along the same lines, while we found no statistical evidence for a significant cannabis exposure effect, we cannot rule out the possibility that significant effects may be found in larger samples, in other contexts (e.g. in a state with cannabis legalization), or cases of sustained heavy use. While the cannabis use index used here captured frequency and amount of use, specific information on delta-9-tetrahydrocannabinol concentration levels was not assessed as part of our interview-based self-report assessment.

Using a quasi-experimental design to leverage between- and within-pair differences in alcohol, cannabis, and nicotine use during emerging adulthood to separate risk and environmental exposure influence, the present study extending our prior pilot study (Wilson et al., 2018) provides evidence that, for women, the lower hippocampal volume appears to reflect both a premorbid substance-related familial risk characteristic and the deleterious consequences of nicotine and alcohol exposure on the still-developing young adult hippocampus. While replication is needed, these sex-specific potential exposure and risk effects have important public health implications regarding etiological models of substance use, targeted preventions, and public health policy. Efforts informed by this work could focus on (a) public messaging alerting young adults to the potential risk for the insult that substance use may have on key brain regions (e.g. the hippocampus) particularly in women, and (b) targeting individuals with this premorbid risk characteristic for preventative efforts.

## Supplementary Material

Supplement

## Figures and Tables

**Table 1.** Descriptive statistics

	Total sample		Women		Men	
Measure	Mean (s.d.)	Range	Mean (s.d.)	Range	Mean (s.d.)	Range
Hippocampal volume (mm^3^)	8256.84 (812.84)	6272.60– 10649.80	7950.15 (715.01)	6272.60–9795.50	8683.18 (747.13)	6969.50– 10649.80
Substance use composite	0.00 (0.83)	−1.56–2.11	−0.25 (0.76)	−1.56–1.94	0.35 (0.79)	−1.56–2.11
Drink Index	2.79 (0.97)	0.00–4.75	2.52 (0.92)	0.00–4.50	3.17 (0.91)	0.00–4.75
Cannabis Index	1.73 (1.76)	0.00–5.00	1.30 (1.57)	0.00–5.00	2.31 (1.85)	0.00–5.00
Cigarettes per day	0.95 (1.13)	0.00–4.00	0.67 (1.04)	0.00–4.00	1.32 (1.14)	0.00–4.00

*Note*: Unadjusted hippocampal volume is reported in this table. All substance use scores were greater for men compared to women (all 95% CIs contrasting men and women did not contain zero).

**Table 2.** Sex-specific individual-level phenotypic associations between total hippocampal volume and substance use phenotypes

Model	Beta [95% CI]	s.e.
Drink Index		
Women	**−124.70 [−189.03 to −56.31]**	**34.18**
Men	6.57 [−97.28 to 106.77]	51.39
Cannabis Index		
Women	**−47.59 [−88.16 to −2.04]**	**22.05**
Men	−12.62 [−58.32 to 35.73]	23.97
Cigarettes per day		
Women	**−120.09 [−182.42 to −56.88]**	**32.19**
Men	−35.47 [−113.17 to 39.21]	38.65

*Notes*: Significant effects are in bold, determined by the nonparametric bootstrap 95% CI around the unstandardized beta estimate not overlapping with zero. CI = confidence interval; s.e. = standard error.

**Table 3.** Cotwin control analysis models between hippocampus volume and substance use composite, drink index, cannabis index, and cigarettes per day scores in women

	Between-pair effect		Within-pair effect	
Model	Beta [95% CI]	s.e.	Beta [95% CI]	s.e.
Substance use composite	**−182.50 [−333.34 to −40.28]**	**72.81**	**−195.62 [−316.37 to −64.60]**	**63.85**
Drink Index	**−200.30 [−326.87 to −76.06]**	**63.62**	**−89.77 [−175.14 to −7.54]**	**42.41**
Cannabis Index	−36.93 [−108.02 to 38.91]	36.89	−54.51 [−114.71 to 10.17]	31.75
Cigarettes per day	**−124.72 [−232.64 to −18.59]**	**54.10**	**−119.30 [−197.40 to −27.58]**	**43.13**

*Notes:* Significant effects are in bold, determined by the nonparametric bootstrap 95% CI around the unstandardized beta estimate not overlapping with zero. CI = confidence interval; s.e. = standard error.
