# Supplementary material for: Testing the consequences of alcohol, cannabis, and nicotine use on hippocampal volume: a quasi-experimental cotwin control analysis of young adult twins
Source: Psychol Med. Author manuscript; Available in PMC 2023 Oct 1. (PMC10123841; doi:10.1017/S0033291721004682)
Supplement: Supplement [file NIHMS1758862-supplement-Supplement.docx]

## Supplemental Information

Table S1. Items used in the drink index, cannabis index, and cigarettes per day measures.

|  | Alcohol/Cannabis |  | Alcohol | | |  | Cannabis |  | Nicotine |
| --- | --- | --- | --- | --- | --- | --- | --- | --- | --- |
| Score | Frequency |  | Amount  (# of drinks) | Intoxications | Max Drinks |  | Amount  (# of uses) |  | Cigarettes per day |
| 0 | None |  | 0 | 0 | 0 |  | 0 |  | None |
| 1 | Less than once per year to less than once a month |  | 1 ­– 3 | 1 – 5 | 1 – 3 |  | 1 – 4 |  | 1 – 2 |
| 2 | 1-3 times per month |  | 4 – 6 | 6 – 10 | 4 – 6 |  | 5 – 30 |  | 3 – 9 |
| 3 | 1-4 times per week |  | 7 – 10 | 11 – 20 | 7 – 10 |  | 31 – 100 |  | 10 (half a pack) – 19 |
| 4 | Nearly every day to  once a day |  | 11 – 20 | 21 – 50 | 11 – 20 |  | 101 – 400 |  | ≥ 20 (a pack or more) |
| 5 | Two or more times a day |  | 21 – 29 | 51 – 149 | 21 – 29 |  | ≥ 401 |  | N/A |
| 6 | N/A |  | ≥ 30 | ≥ 150 | ≥ 30 |  | N/A |  | N/A |

Note: Cannabis amount and alcohol/cannabis frequency items are on a 0 – 5 scale, cigarettes per day is on a 0 – 4 scale, and the remaining items are on a 0 ­– 6 scale.

**Supplemental Results**

*Do hippocampal volume-substance use associations differ between left and right hippocampus?*

To test whether the substance use-hippocampal volume associations differed by hemisphere, the main models were recomputed to include an interaction term between hemisphere (effect coded for right hemisphere) and each substance use phenotype. As shown in Table S2, right hippocampal volume was larger than left, but all interactions between hemisphere and substance use were small in magnitude and the 95% confidence intervals overlapped with zero. This provides evidence that the observed substance use effects were statistically equivalent across left and right hemispheres and support our use of the total hippocampal volume measure in the main analyses.

Table S2. Hippocampal volume: interaction between hemisphere and substance use phenotypes.

|  | Women | |  | Men | | |
| --- | --- | --- | --- | --- | --- | --- |
| Model | Beta  [95% CI] | SE |  | Beta  [95% CI] | | SE |
| Substance use composite |  |  |  |  | |  |
| Hemisphere | **63.33**  **[44.95, 81.44]** | **9.27** |  | **54.97**  **[30.36, 81.58]** | | **12.92** |
| Substance use  composite | **-93.32**  **[-133.40, -50.66]** | **21.12** |  | -14.25  [-68.77, 34.54] | | 25.82 |
| Interaction | 4.44  [-19.69, 24.90] | 11.27 |  | 0.76  [-32.59, 28.30] | | 15.12 |
| Drink Index |  |  |  |  | |  |
| Hemisphere | 45.27  [-2.01, 95.36] | 25.21 |  | 42.98  [-38.12, 131.76] | | 43.03 |
| Drink Index | **-58.37**  **[-87.57, -27.21]** | **15.27** |  | 5.10  [-39.46, 52.14] | | 23.17 |
| Interaction | 6.73  [-12.10, 24.44] | 9.42 |  | 3.87  [-22.89, 29.52] | | 13.08 |
| Cannabis Index |  |  |  |  |  |  |
| Hemisphere | **53.23**  **[31.54, 76.93]** | **11.50** |  | **29.52**  **[13.51, 88.17]** | | **18.95** |
| Cannabis Index | **-24.55**  **[-43.87, -3.09]** | **10.25** |  | 49.37  [-27.86, 14.59] | | 10.81 |
| Interaction | 6.88  [-4.48, 17.48] | 5.59 |  | -6.74  [-10.56, 14.45] | | 6.37 |
| Cigarettes per day |  |  |  |  |  |  |
| Hemisphere | **67.64**  **[47.84, 88.87]** | **10.40** |  | **63.07**  **[26.18, 97.16]** | | **18.30** |
| Cigarettes per day | **-59.89**  **[-87.97, -30.18]** | **14.90** |  | -14.37  [-50.27, 18.74] | | 17.65 |
| Interaction | -8.09  [-26.02, 7.16] | 8.37 |  | -5.92  [-27.41, 13.66] | | 10.44 |

*Notes*: The hemisphere term is coded for right hemisphere. Significant effects are in bold, determined by the nonparametric bootstrap 95% confidence interval around the unstandardized beta estimate not overlapping with zero. CI = confidence interval; SE = standard error.

**Supplemental Figure**

**Figure S1.** Distribution of the observed within-pair and between-pair scores for the phenotypes used in the cotwin control (CTC) analyses. Note that only the women twin pairs included in the CTC analyses (see main text) are plotted here. The *x*-axis represents the scores for the within-pair and between-pair scores (see the Methods section for further details); the *y*-axis represents the count of individuals for each score.
